# Hepatitis A seroprevalence, vaccination status and demographic determinants in children and adolescents in Germany, 2014–2017, a population-based study

**DOI:** 10.1038/s41598-023-36739-4

**Published:** 2023-06-16

**Authors:** Julia Enkelmann, Ronny Kuhnert, Klaus Stark, Mirko Faber

**Affiliations:** 1 Department of Infectious Disease Epidemiology, Robert Koch Institute, Berlin, Germany; 2 Department of Epidemiology and Health Monitoring, Robert Koch Institute, Berlin, Germany

**Keywords:** Diseases, Risk factors

## Abstract

Children play an important role in hepatitis A virus (HAV) transmission but, due to frequent asymptomatic or mild courses, these infections are underrecognized in routine surveillance. Here, we analyzed hepatitis A (HA) seroprevalence, vaccination status and demographic determinants and estimated previous HAV infections in a cross-sectional population-based study of children and adolescents with residence in Germany 2014–2017, performing weighted univariable and multivariable logistic regression. Of 3567 participants aged 3–17 years, serological results were available for 3013 (84.5%), vaccination records for 3214 (90.1%) and both for 2721 (76.3%). Of 2721 with complete results, 467 (17.2%) were seropositive, thereof 412 (15.1%) with and 55 (2.0%) without previous HA vaccination, indicating previous HAV infection. Seropositivity was associated with age, residence in Eastern states, high socioeconomic status and migration background with personal migration experience. Participants with migration background and personal migration experience also had the highest odds ratios for previous HAV infection. Germany remains a country with very low HA endemicity. The current vaccination recommendations focusing on individuals with a high risk for HAV exposure (e.g. travelers to endemic countries) or severe disease appear appropriate. Migration and travel patterns as well as the endemicity in other countries influence the domestic situation, warranting further monitoring.

## Introduction

Hepatitis A (HA) is caused by an infection with hepatitis A virus (HAV), which is transmitted through the fecal–oral route. In adults, the majority of infections lead to an acute symptomatic hepatitis, with the risk of severe disease increasing with age and preexisting liver disease. Young children typically remain asymptomatic or present with only mild symptoms, thereby contributing to HAV dissemination while being underrecognized in routine surveillance data. Since 2012 annual incidences of notified HA in Germany have ranged between 0.7 and 1.5 per 100,000 population^1^.

HAV infection is vaccine-preventable. The National Standing Committee on Vaccination is recommending HA vaccination for individuals at higher risk of HAV exposure and those with risk factors for severe disease since 1993^2^, currently including people traveling to endemic countries^3^, people with increased professional or sexual exposure, with repeated contact to blood products or with liver disease and residents of psychiatric or comparable institutions^4^. In 1998, Saxony, one of the 16 federal states in Germany, implemented a universal HA vaccination recommendation for children older than 13 months^5,6^.

As part of the nationwide health monitoring, the representative German Health Interview and Examination Survey for Children and Adolescents (KiGGS study) was implemented. A baseline study was conducted between 2003 and 2006 and found an overall crude HAV seroprevalence of 14%. Of those, 11% had a documented previous HA vaccination and the remaining 3% were attributed to previous HAV infection^7^. The most recent wave 2 was carried out between 2014 and 2017. We describe HA seroprevalence and vaccination status, estimate the prevalence of previous HAV infections of children participating in KiGGS wave 2, and compare the results to those of the baseline study. Besides providing an overview of the most current representative data on HA epidemiology among children and adolescents in Germany, we aim to identify groups at higher risk of infection that could benefit from public health interventions, e.g. focused HA vaccination recommendations.

## Results

**Figure 1:**
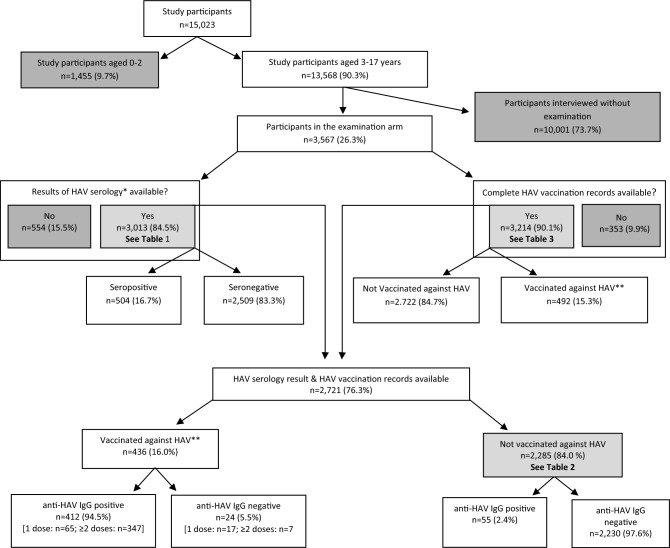
Flow chart of KiGGS 2 study: study participants, number of participants providing serum samples for HAV testing, HA vaccination records and anti-HAV-IgG-antibody prevalence according to HA vaccination status. *Anti-HAV-IgG-antibodies, **Received ≥ 1 dose of HA vaccine. Dark grey = excluded; light grey = additional information available in the referenced tables.

Of 37,420 invited children and adolescents, 15,023 (40.1%) participated in wave 2 of the KiGGS study (Fig. 1). Of 13,568 participants aged ≥ 3 years, 3567 (26.3%) took part in the examination arm, which included the review of vaccination records and HA serology (anti-HAV-Immunoglobin-G (IgG)-antibodies in serum). Complete vaccination records were provided by 3214 (90.1%) and anti-HAV-IgG antibody results were available for 3013 (84.5%). The proportion of participants providing a blood sample increased with age. Both, HA serology result and HA vaccination status, were available for 2721 (76.3%) children and adolescents in the examination arm, thereof 467 (17.2%) were seropositive: 412 (15.1%) with and 55 (2.0%) without a documented previous HA vaccination, thus indicating previous HAV infection.

### Overall seroprevalence

**Figure 2:**
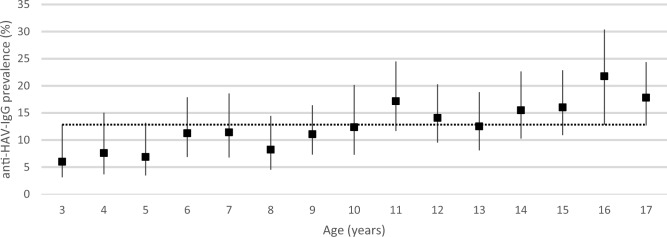
Estimated anti-HAV IgG prevalence by age, KiGGS study wave 2, Germany 2014–2017 (n = 3013). Error bars indicate 95% CI. The dashed line depicts the mean weighted seroprevalence overall (12.83%).

Of 3013 participants with available HA serology results, 504 (16.7%) were anti-HAV-IgG positive. Using the appropriate sample weights, the population prevalence of anti-HAV-IgG among children and adolescents aged 3–17 years in Germany was estimated at 12.8% (95% confidence interval (CI) 10.9–15.0%), increasing significantly with age (Table 1, Fig. 2). In a weighted multivariable analysis including age, sex, place of residence, socioeconomic status (SES) and migration background, a significantly higher HAV seroprevalence was observed in children and adolescents with high SES, two-sided migration background with personal migration experience and those residing in states in Eastern Germany, most notably Saxony (Table 1). The overall seroprevalence did not differ significantly between males and females, or by urbanity.

**Table 1:** Overall HAV seroprevalence: estimated population prevalence of anti-HAV-IgG-antibodies in children aged 3–17 years in Germany, 2014–2017, by demographic characteristics and results of univariable and multivariable analyses. All study participants with available HA serology results were included in the analysis (n = 3013).

Characteristic	n	Weighted seroprevalence	Weighted univariable analysis	Weighted multivariable analysis^b^
		% (95% CI)	Odds ratio (95% CI)	Adjusted Odds ratio (95% CI)
All participants with HA serology results	3013	12.83 (10.92–15.02)		
Age (yearly)	3013		**1.09 (1.05–1.12)**	**1.11 (1.07–1.15)**
Sex				
Male	1487	12.27 (10.05–14.90)	Reference	Reference
Female	1526	13.43 (11.07–16.19)	1.11 (0.86–1.43)	1.09 (0.83–1.45)
Place of residence				
West Germany including Berlin	2099	10.10 (8.44–12.06)	Reference	Reference
East Germany excluding Saxony	607	13.03 (10.08–16.67)	1.39 (0.97–1.98)^a^	**1.60 (1.11–2.31)**
Saxony	307	64.47 (56.35–71.84)	**18.76 (12.40–28.38)**^**a**^	**20.90 (13.39–32.62)**
Urbanity (inhabitants)				
< 5000	618	14.49 (9.70–21.11)	Reference	
5000–< 20,000	866	12.11 (8.79–16.46)	0.81 (0.46–1.45)	
20,000–< 100,000	839	13.68 (10.36–17.86)	0.94 (0.54–1.63)	
≥ 100,000	690	11.74 (8.45–16.08)	0.78 (0.44–1.41)	
Socioeconomic status				
Low	426	14.04 (10.21–19.00)	1.17 (0.79–1.73)	1.10 (0.72–1.65)
Medium	1782	12.23 (10.08–14.77)	Reference	Reference
High	698	13.67 (11.06–16.77)	1.14 (0.89–1.46)	**1.37 (1.03–1.83)**
“Missing”	107			
Migration background				
Non-migrant	2248	12.65 (10.49–15.18)	Reference	Reference
One-sided	266	10.27 (6.71–15.41)	0.79 (0.49–1.28)	1.09 (0.66–1.82)
Two-sided, living in Germany since birth	320	13.34 (8.92–19.48)	1.06 (0.66–1.71)	1.45 (0.89–2.36)
Two sided with personal migration experience	94	25.07 (15.24–38.37)	**2.31 (1.21–4.42)**	**2.59 (1.28–5.23)**
“Missing”	85			

^a^Adjusted for age.

^b^Results of the final multivariable model after stepwise backwards elimination of variables with p > 0.05 (except age and sex, which were kept irrespective of p-values).

Statistically significant results are highlighted in bold.

### HAV infections

Of 2285 unvaccinated participants, 55 (2.4%) had detectable anti-HAV IgG-antibodies, indicating previous HAV infection. This corresponds to a (weighted) population prevalence of previous HAV infection of 3.1% (95% CI 2.3–4.3) in unvaccinated children aged 3–17 years in Germany.

In weighted multivariable logistic regression including age, sex, place of residence, SES and migration background, the prevalence of previous HAV infection was significantly higher in unvaccinated participants residing in Saxony and those with two-sided migration background with personal migration experience (Table 2). Prevalence of previous HAV infection increased with age and was higher in males, participants residing in middle-sized or large cities and those with two-sided migration background living in Germany since birth, but those differences were not statistically significant. Low SES was associated with previous HAV infection in univariable, but not in multivariable analysis.

**Table 2:** HAV infections: estimated prevalence of anti-HAV IgG-antibodies in unvaccinated children aged 3–17 years in Germany, 2014–2017, thus indicating previous infection, by demographic characteristics and results of univariable and multivariable analyses. All unvaccinated participants with complete vaccination records and HA serology results were included (n = 2285).

Characteristic	n	Weighted seroprevalence	Weighted univariable analysis	Weighted multivariable analysis^b^
		% (95% CI)	Odds ratio (95% CI)	Adjusted Odds ratio (95% CI)
All unvaccinated participants with complete vaccination records with HA serology results	2285	3.13 (2.25–4.33)		
Age (yearly)			1.02 (0.94–1.11)	1.00 (0.91–1.10)
Sex				
Male	1145	4.08 (2.60–6.33)	Reference	Reference
Female	1140	2.12 (1.31–3.41)	0.51 (0.25–1.04)	0.55 (0.26–1.16)
Place of residence				
West Germany including Berlin	1684	3.05 (2.11–4.40)	Reference	Reference
East Germany excluding Saxony	500	1.73 (0.88–3.36)	0.56 (0.26–1.23)^a^	0.70 (0.29–1.68)
Saxony	101	7.48 (3.02–17.32)	2.62 (0.94–7.26)^a^	**3.49 (1.12–10.88)**
Urbanity (inhabitants)				
< 5000	445	2.38 (1.26–4.44)	Reference	
5000–< 20,000	669	2.60 (1.42–4.71)	1.10 (0.45–2.68)	
20,000–< 100,000	645	3.52 (1.90–6.42)	1.50 (0.61–3.69)	
≥ 100,000	526	3.65 (1.96–6.70)	1.56 (0.63–3.86)	
Socioeconomic status				
Low	329	6.31 (3.37–11.52)	**3.37 (1.47–7.75)**	
Medium	1362	1.96 (1.22–3.14)	Reference	
High	541	3.46 (1.77–6.64)	1.79 (0.76–4.24)	
“Missing”	53			
Migration background				
Non-migrant	1738	2.03 (1.31–3.13)	Reference	Reference
One-sided	204	1.98 (0.74–5.18)	0.97 (0.32–2.95)	1.05 (0.34–3.21)
Two-sided, living in Germany since birth	243	3.37 (1.35–8.20)	1.69 (0.59–4.80)	1.92 (0.67–5.56)
Two sided with personal migration experience	57	25.77 (13.23–44.14)	**16.76 (6.42–43.76)**	**16.95 (6.58–43.64)**
“Missing”	43			

^a^Adjusted for age.

^b^Results of the final multivariable model after stepwise backwards elimination of variables with p > 0.05 (except age and sex, which were kept irrespective of p-values).

Statistically significant results are highlighted in bold.

Of 55 participants with presumed previous HAV infection, 21 (38.2%) had a migration background, thereof 9 with personal migration experience. Their parents had been living in Germany for a median of 7 years (range 1–10), suggesting that the respective children had lived a median of 5 years (range 4–15) outside of Germany. Their home countries were in Arabic countries in Asia (n = 4), Africa (n = 2), non-Mediterranean countries in Europe (n = 2) and Mediterranean countries in Europe (n = 1); 4 had entered Germany as refugees seeking asylum.

The parents of the 12 children with migration background that had lived in Germany since birth had roots in non-Mediterranean countries in Europe (n = 4), Mediterranean countries in Europe (n = 3), Turkey (n = 2), non-Arabic countries in Asia (n = 2) and Arabic countries in Asia (n = 1) and had lived in Germany for a median of 23 years (range 8–40 years, information available for n = 11).

### Hepatitis A vaccinations

Of 3,214 participants that provided complete vaccination records, 492 (15.3%) had received at least one dose of HA vaccine prior to the study, corresponding to a population prevalence of HA vaccination of 11.9% (10.1–14.1). The number of doses received ranged from 1 to 6 (1 dose: 19.5%, 2 doses: 65.9%, 3 doses: 13.0%, remaining 1.6% 4–6 doses). Information sufficient to determine completeness of the basic immunization course was available for 481 participants: it was complete in 368 (76.5%) and incomplete in 113 (23.5%), including 29 children who had received a dose of monovalent vaccine in the year preceding the investigation. The population prevalence of complete HA vaccination was estimated at 8.5% (95% CI 7.0–10.3) for children aged 3–17 years in Germany. The majority of participants had exclusively received monovalent vaccines (n = 391), followed by HA/hepatitis B (HB) combination vaccines (n = 76) and a mix of both (n = 12). For the remaining participants the type of vaccine received was not specified for at least 1 dose (n = 13).

The odds of previous HA vaccination adjusted for age was 21.2-times (95% CI 13.1–34.4, p < 0.001) higher in children residing in Saxony compared to participants residing in West Germany.

Population prevalence of HA vaccination excluding Saxony was estimated at 9.5% (95% CI 8.0–11.2), and positively associated with age in multivariable logistic regression (Table 3). There was a trend towards a higher prevalence of previous HA vaccination with increasing SES (Table 3).

**Table 3:** Hepatitis A vaccination: Estimated weighted prevalence of HA vaccination in children aged 3–17 years in Germany, 2014–2017, by demographic characteristics, univariable and multivariable analyses. Participants with complete vaccination records and place of residence other than Saxony (state with universal vaccination recommendation) were included (n = 2875).

Characteristic	n	Weighted prevalence	Weighted univariable analysis	Weighted multivariable analysis^b^
		% (95% CI)	Odds ratio (95% CI)	Odds ratio (95% CI)
Total with vaccination records	2875	9.47 (8.01–11.17)		
Age (yearly)			**1.15 (1.11–1.19)**	**1.15 (1.11–1.19)**
Sex				
Male	1417	8.75 (6.99–10.91)	Reference	Reference
Female	1458	10.23 (8.08–12.88)	1.19 (0.84–1.68)	1.20 (0.85–1.70)
Place of residence				
West Germany including Berlin	2194	9.38 (7.80–11.23)	Reference	
East Germany excluding Saxony	681	10.34 (7.55–14.01)	1.18 (0.78–1.78)^a^	
Urbanity (inhabitants)				
< 5000	519	9.30 (5.72–14.77)	Reference	
5000–< 20,000	836	8.74 (6.44–11.76)	0.93 (0.50–1.74)	
20,000–< 100,000	865	11.22 (8.41–14.83)	1.23 (0.67–2.28)	
≥ 100,000	655	8.35 (6.01–11.47)	0.89 (0.47–1.67)	
Socioeconomic status				
Low	417	8.13 (5.26–12.36)	0.86 (0.53–1.41)	
Medium	1705	9.32 (7.58–11.41)	Reference	
High	681	11.41 (8.86–14.57)	1.25 (0.90–1.74)	
“Missing”	72			
Migration background				
Non-migrant	2132	9.55 (7.90–11.50)	Reference	
One-sided	283	9.34 (6.10–14.05)	0.98 (0.62–1.55)	
Two-sided, living in Germany since birth	338	10.34 (6.98–15.05)	1.09 (0.68–1.76)	
Two sided with personal migration experience	72	8.00 (3.57–16.97)	0.82 (0.36–1.91)	
“Missing”	50			

Received at least 1 dose of HAV vaccine.

^a^Adjusted for age.

^b^Results of the final multivariable model after stepwise backwards elimination of variables with p > 0.05 (except age and sex, which were kept irrespective of p-values).

Statistically significant results are highlighted in bold.

In 24 participants with a history of HA vaccination, no anti-HAV-IgG antibodies were detected: thereof 6 (25%) had received a complete course of HA vaccination (3 × regimen with monovalent vaccine, 3 × regimen with HA/HB combination vaccine), 17 (71%) had only received 1 dose (15 monovalent vaccine, 2 combination vaccine) and 1 person had received 2 doses of a non-specified vaccine. This corresponds to an antibody prevalence of 98.4% in fully vaccinated participants and 85.0% of participants with incomplete HA vaccination.

## Discussion

This representative cross-sectional study showed that approximately 13% of children aged 3–17 years residing in Germany 2014–2017 were anti-HAV IgG positive, approximately 12% were vaccinated against HA (≥ 1 dose). Among unvaccinated, approximately 3% were seropositive, indicating previous infection. In the baseline study 2003–2006, in which > 13,000 children were examined, seropositivity in children aged 3–17 in Germany was, too, estimated at 13%, previous vaccination slightly lower at 11% and previous infection among unvaccinated individuals at 3%^7^.

We found that most children had acquired immunity through vaccination and only a minority through infection. Seropositivity was associated with age, residence in Eastern German states, especially Saxony, high SES and two-sided migration background with personal migration experience. Seropositivity without a documented vaccination (indicating previous HAV infection) was associated with residence in Saxony and two-sided migration background with personal migration experience. Vaccination was only associated with age.

Comparable to the baseline study, we found an association of previous HAV infection with two-sided migration background, but in the current analysis, we further subdivided into those living in Germany since birth and those with personal migration experience. Both had higher odds of previous HAV infection than participants without migration background, but the difference was only statistically significant in case of personal migration experience, suggesting that the main association with infection was previously living abroad. The World Health Organization classifies the level of HA endemicity based on age-specific HA seroprevalence in the general population as high (≥ 90% by age 10), intermediate (≥ 50% by age 15 with < 90% by age 10), low (≥ 50% by age 30 with < 50% by age 15) and very low (< 50% by age 30)^8^. As most HAV infections in regions with high HA endemicity occur during early childhood^9^, children that migrated from or through these regions may have experienced HAV infection prior to arrival in Germany.

Compared to tourists, children visiting friends and relatives (VFR) in countries with higher HA endemicity may furthermore have an increased risk of HAV exposure due to longer travel durations and living conditions and food consumption approximating those of the local population^10^. Enhanced HA surveillance revealed that most imported cases in Germany 2007–2008 occurred in young people with migration background who had VFR in their ancestral countries (at that time most commonly Turkey or countries in the region of former Yugoslavia) and the majority stayed in accommodations other than hotels^11^. Even in the absence of travel, HAV strains of cases with migration background clustered with imported strains of the respective region, while strains from autochthonous cases without migration background were very diverse^11^, suggesting that having a migration background could be an independent risk factor for HAV infection, e.g. through contact with returning travelers or guests from HAV endemic countries in the household, or consumption of contaminated food imported from these regions.

Yet, the spectrum of ancestral countries of children with migration background in Germany is constantly changing. During the study period e.g. many people migrating to and seeking asylum in Germany were arriving from Arabic countries in Asia^12,13^ with high HA endemicity^14^. Currently (2022), Germany is receiving a large number of people fleeing the war in Ukraine, a country with previously intermediate HA endemicity in rural and low endemicity in urban areas. However, degradation of sanitary conditions related to destruction of critical infrastructure poses a risk for HAV transmission locally and during migration^17,18^. Historically a high proportion of people with migration background in Germany had roots in Turkey, Mediterranean countries and regions of former Yugoslavia, in many of which HA endemicity has decreased in the last decade. Turkey and Greece for example have implemented routine HA vaccination programs in 2012 and 2008, respectively, with high uptake^15,16^, resulting in a lower risk of HAV acquisition and importation to Germany. Effects of changing migration patterns and circumstances in ancestral countries warrant further epidemiological investigation and monitoring.

The association of previous HAV infection and residence in Saxony- although compatible with results of the baseline study, in which higher odds of previous infection were observed in the central East region, which includes Saxony^7^—is an unexpected finding and has to be interpreted with caution. According to notification data, cumulative HA incidence 2014–2017 was lowest in Saxony and with a comparable proportion of the population having a migration background, similar uptake of child care, travel and food consumption to the surrounding Eastern states, we are not aware of reasons why children residing in Saxony should be at higher risk of HAV infection. However, as Saxony is the only state in Germany with a universal vaccination recommendation for HAV^5^, overestimation of previous HAV infections due to undocumented HAV vaccinations, may have disproportionately affected estimates for Saxony, where vaccination rates are much higher than in the rest of the country. A serological method distinguishing between vaccine-induced immunity and natural infection could be used in future studies to investigate this further^19,20^.

Children residing in Saxony had by far the highest prevalence of HA vaccination, as expected with the universal HA vaccination policy in this state. The association with increasing age was also expected due to cumulation over time.

Unlike in the baseline study, there was no significant association between high SES and HA vaccination^7^, but the prevalence of previous HA vaccination did again increase with increasing SES. This is in line with results of the German health interview and vaccination survey for adults (DEGS1, 2008–2011), that found similar overall seroprevalences in participants with high and low SES and higher vaccination rates in those with high SES, suggesting that seropositivity in adults with low SES was more likely due to previous HAV infection, while those with high SES were more likely vaccinated^21,22^. This could indicate better information of families with higher SES about HA vaccination opportunities, or different travel patterns and higher uptake of (pre-travel) medical consultations including HA vaccination.

Of participants with documented HA vaccination, < 2% of fully vaccinated and 15% of those with incomplete vaccination were seronegative. This is in line with findings that most healthy children and adolescents seroconvert after the first HA vaccine dose (typically ≥ 95% using monovalent vaccines) and no response after 2 doses is rare^23^. The humoral response can be blunted e.g. in immunocompromised individuals and with high body mass index^3,23,24^. Follow-up investigations in Panama- a country with a 1-dose HA vaccination program- showed that after 10 years, antibodies persisted in > 70% of young people^25^. In Germany, depending on the vaccine used, 2 (monovalent) or 3 doses (combination with hepatitis B) are recommended for longterm-protection^4^.

Since the completion of KiGGS wave 2, HA outbreaks in Germany have occurred among men who have sex with men^26^, people seeking asylum living in mass accommodation^27^ and returning travelers^28^. In addition, several foodborne outbreaks were observed among people without travel history implicating, e.g. imported dates^28^ and frozen berries^29^ as vehicles. It is unknown if this has increased awareness about HAV infection and if it had an impact on vaccination rates in children. In view of the many factors potentially affecting the HAV epidemiology in Germany (migration patterns, HAV epidemiology in regions frequently visited by people living in Germany, food safety, vaccination uptake), a follow-up study would be useful to monitor trends.

Our study has some limitations. Due to the cross-sectional nature of the study a cohort effect cannot be excluded. Previous HAV infections may be overestimated due to non-consideration of seronegative participants with incomplete, illegible and no vaccination records. The number of seropositive unvaccinated participants was very low, therefore associations with previous infections need to be interpreted with caution. Some trends did not reach statistical significance in multivariable analysis. We cannot exclude that the examination arm of the study was underpowered, possibly compounded by missing serological data or vaccination records. The applied weighting factors only ensure representativeness in terms of age, sex, urbanity of place of residence, citizenship and parent’s level of education.

## Conclusion

This study provides insights into the more recent developments of the epidemiology of HA among children living in Germany and constitutes an important basis for further public health recommendations. Despite a slightly higher population fraction of vaccinated individuals in 2014–2017, estimates of seroprevalence and previous HAV infection are similar to the results of the baseline study conducted 2003–2006^7^. In conjunction with HA seroprevalence rates from a representative study among adults in Germany 2008–2011^21^, the results confirm Germany’s status as a country with very low HA endemicity. The current vaccination recommendations focusing on risk groups such as travelers to endemic countries and those at higher risk for severe disease seem appropriate.

Medical providers should inform families planning to travel to HA endemic regions about the vaccination recommendation, especially targeting children with migration background VFR in countries with higher HA endemicity, as VFR-travel may correspond to an increased HAV exposure. These public health issues should be addressed by publications and other information activities targeted at the medical providers.

Although travel-vaccinations are not generally reimbursed, almost all German health insurance providers reimburse costs of travel-related HA-vaccinations upon request^30^. Simplifying the reimbursement process would reduce barriers for provision and might also increase the uptake of recommended HA vaccinations. This would not only benefit individuals but could also reduce secondary transmission especially in child care facilities and schools in Germany, where—as shown in this study—most children are susceptible to HAV infection.

The recent autochthonous foodborne outbreaks highlight that certain imported food products available in countries with very low endemicity (such as Germany) can also be a relevant source of HAV infection (e.g. frozen berries, dates). Continuing HA surveillance and expanding molecular surveillance is crucial to enable public health authorities to investigate and rapidly terminate transmission chains in a largely susceptible population.

## Methods

The KiGGS study is part of the national health monitoring and provides representative cross-sectional data for children and adolescent aged ≤ 17 years with permanent residence in Germany. Wave 2 was conducted between September 2014 and June 2017. The study was approved by the Federal Commissioner for Data Protection and Freedom of Information in Germany and by the ethics committee of Hannover Medical School (No. 2275-2014) and was performed in accordance with relevant guidelines and regulations. Participants, their parents and/or legal guardian provided written consent.

The detailed methodology is described elsewhere^31–33^. Briefly summarized, participants were recruited through a two-stage cluster sampling. First, 167 municipalities that were selected during the baseline study to reflect Germany’s regional structure in terms of state and type of municipality were used as sample points^34^. Then, for each sample point, children were randomly selected from the population registries and invited to participate.

Interview data were collected for all participants. A randomly selected subset of children ≥ 3 years underwent additional examinations, including physical examination, laboratory tests (including HA serology) and structured review of vaccination records.

Qualitative anti-HAV-IgG-antibodies were measured in serum using HAV-chemiluninescence-microparticle immunoassay (CMIA) on the ARCHITECT (Abbot Diagnostics, Land). Samples with a sample/cut-off (S/CO) of < 1 were considered as non-reactive (negative), samples with S/CO ≥ 1.0 were considered reactive (positive).

The date(s) of HA vaccination, type of vaccine and number of HA vaccination doses received were recorded. Participants who had received at least 1 dose of HA vaccine, were considered as vaccinated, as most healthy children and adolescents already develop a humoral antibody response after the first vaccination. The vaccination course was considered complete after 3 doses of HA/HB combination vaccine or 2 doses of monovalent vaccine.

The SES was categorized as low, intermediate or high based on information provided by the parents/legal guardian on education, income and occupation. The methodology was described elsewhere^35^.

The migration background was categorized into no migration background, one-sided or two-sided. The detailed methodology was described elsewhere^33,36^. If both parents were born abroad (or held nationalities other than German) or if the participant and at least one parent were born abroad this was considered a two-sided migration background. We further subdivided participants with two-sided migration background into those with personal migration experience and those living in Germany since birth.

The urbanity of the place of residence was categorized according to population size: < 5000 inhabitants, 5000–< 20,000 inhabitants, 20,000–< 100,000 inhabitants, > 100,000 inhabitants.

The region of residence was categorized in East and West Germany according to the sampling plan.

Unlike the rest of Germany, Saxony has implemented a universal vaccination policy for HAV since 1998, therefore we report the results for this state separately. East Germany (excluding Saxony) includes: Mecklenburg-Western-Pomerania, Brandenburg, Saxony-Anhalt and Thuringia. West Germany includes: Schleswig–Holstein, Hamburg, Bremen, Lower Saxony, North Rhine-Westphalia, Hesse, Rhineland-Palatinate, Saarland, Baden-Wuerttemberg, Bavaria and Berlin.

Statistical analysis was performed using STATA version 17.0. Calculations were carried out after applying a weighting factor adjusting for deviation of the subgroup of participants providing a serum sample from the population structure regarding regional structure (rural/urban), age, sex, state, citizenship and the parents’ level of education. For subgroup-analyses to estimate prevalence of infection and previous HA vaccination the applied weighting factors were adjusted to the subgroup of interest accordingly.

The following outcomes were analyzed in separate logistic regression models:

1. HAV seropositivity in participants that provided a blood sample,
2. HAV seropositivity in participants with complete vaccination records without previous HA vaccination, thus indicating previous HAV infection and
3. Prevalence of HA vaccination in participants that provided complete vaccination records. Participants from Saxony were excluded in this analysis, to ensure that effects of the universal vaccination recommendation in this state did not obscure factors associated with HA vaccination in the rest of Germany.

Point prevalences, univariable and multivariable odd ratios (ORs) and their 95% CI were calculated. We considered results to be statistically significant if the 95% CI of the respective OR did not include the value 1. ORs were estimated using logistic regression. The reference was set to males residing in Western Germany with intermediate SES and no migration background. The model for multivariable logistic regression was built, starting with inclusion of age, sex, region of residence, urbanity of residence, SES and migration background as independent variables. Then stepwise backward elimination was performed, starting with the least significant parameter. Submodels were compared using the F-test (adjusted Wald test). Elimination of parameters was continued until no parameters with a p-value of > 0.05 remained in the model, except age and sex, which were kept in the model irrespective of p-values. Then, all eliminated parameters were reintroduced separately into the final model, to test (again using the F-test) if they statistically significantly improved the final model.

## Data Availability

A scientific use file of data of wave 2 of the KiGGS study is available upon request at https://www.kiggs-studie.de/ergebnisse/kiggs-welle-2/scientific-use-file.html.
